# A Dissection Within a Dissection: Iatrogenic Left Main Coronary Artery Dissection Complicating Spontaneous Coronary Artery Dissection-Related Myocardial Infarction

**DOI:** 10.7759/cureus.111162

**Published:** 2026-06-19

**Authors:** Laktib Nabil, Selma Saidi, Amer Zabalawi

**Affiliations:** 1 Cardiology Center, Mohammed V Military Teaching Hospital, Rabat, MAR; 2 Department of Cardiology, Hôpital Universitaire Avicenne, Rabat, MAR; 3 Department of Cardiology, Centre Hospitalier Yves Le Foll, Saint-Brieuc, FRA

**Keywords:** coronary intervention, iatrogenic dissection, left main dissection, myocardial infarction, spontaneous coronary artery dissection

## Abstract

Spontaneous coronary artery dissection (SCAD) is an important cause of myocardial infarction in young women. Coronary instrumentation may worsen the dissection, particularly during percutaneous coronary intervention. A 39-year-old woman presented with a posterior ST-segment elevation myocardial infarction due to a Saw type I SCAD of the second marginal artery. During coronary angiography, guidewire manipulation and contrast injection precipitated an acute iatrogenic dissection of the left main coronary artery (LMCA) extending into the left anterior descending and circumflex arteries, resulting in hemodynamic collapse. Emergency stenting of the left main and circumflex arteries restored stable coronary flow. The initial lesion was managed conservatively and showed near-complete healing at three weeks. This case highlights the propensity for iatrogenic complications in SCAD owing to arterial wall fragility, reinforcing the need for a cautious, stepwise interventional approach. In SCAD, early recognition and limiting coronary manipulation are essential. When an LMCA compromise occurs, rapid stenting is lifesaving, while the original dissection lesion should subsequently be managed conservatively.

## Introduction

Spontaneous coronary artery dissection (SCAD) is an increasingly recognized cause of acute coronary syndrome, accounting for approximately 1-4% of cases and up to 22-35% of myocardial infarctions in women younger than 50 years of age [1]. It is strongly linked to arteriopathy and hormonal influences. Its management remains challenging, particularly during coronary intervention, where coronary instrumentation may induce or extend dissection. Contemporary literature indicates that iatrogenic coronary dissection is rare in the general population (≈0.2%), but in patients with SCAD, it may occur in approximately 2% of cases, reflecting the inherent fragility of the affected arterial walls [2].

Procedural complications in SCAD may result from catheter trauma, guidewire passage into a false lumen, balloon inflation, or contrast-induced hydraulic extension in a fragile coronary artery. Such complications can rapidly compromise coronary blood flow and may require urgent revascularization despite the generally conservative management paradigm favored in SCAD.

Left main coronary artery (LMCA) dissection is one of the rarest but most feared complications of coronary angiography and percutaneous intervention. Contemporary reviews generally cite an incidence of <0.1% [3]. Given its potential to abruptly compromise a large myocardial territory, LMCA dissection may result in hemodynamic collapse, arrhythmias, or sudden cardiac death. Owing to its infrequent occurrence, reports describing its mechanisms remain limited, and optimal management is based primarily on expert consensus. Early recognition and prompt restoration of coronary flow are therefore critical determinants of outcome.

We describe a case of abrupt dissection propagation into the LMCA, left anterior descending artery (LAD), and left circumflex artery (LCx) following minimal contrast injection into a spontaneously dissected second obtuse marginal branch. This report examines the mechanisms of iatrogenic coronary dissection in a patient presenting with SCAD, highlighting the complex interplay between spontaneous arterial wall pathology and procedural triggers. It underscores the potentially catastrophic consequences of coronary instrumentation in SCAD and illustrates the challenges of balancing conservative and interventional strategies in this setting. The case further emphasizes the importance of meticulous catheter handling and limited coronary manipulation to reduce the risk of iatrogenic dissection while outlining contemporary management approaches when life-threatening extension occurs.

## Case presentation

A 39-year-old woman presented with acute chest pain associated with a posterior ST-segment elevation myocardial infarction (STEMI). Apart from active smoking, obesity, and use of a levonorgestrel intrauterine device, she had no significant cardiovascular history. No personal or family history suggestive of connective tissue disease or inherited arteriopathy was identified.

On admission, blood pressure was 135/83 mmHg, heart rate was 83 bpm, and physical examination was unremarkable (Killip class I). Electrocardiography demonstrated ST-segment depression in leads V1-V3 with subtle posterior ST-segment elevation on posterior leads (Figure 1).

**Figure 1:**
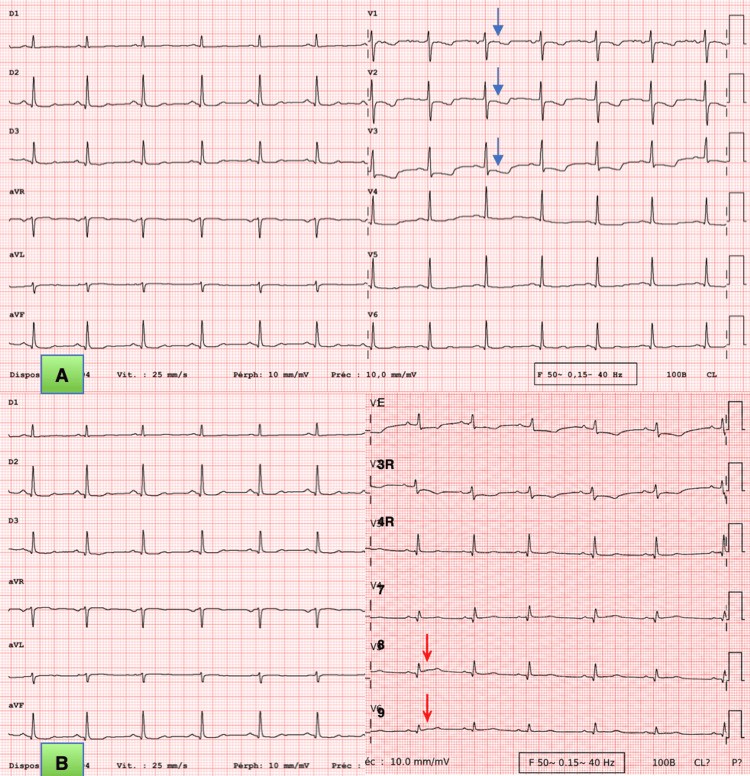
Electrocardiographic findings at presentation (A) Twelve-lead electrocardiogram demonstrating horizontal ST-segment depression in leads V1-V3 (arrows) with an R/S ratio >1 in V2, consistent with posterior STEMI. (B) Posterior leads (V7-V9) demonstrating subtle ST-segment elevation with early Q-wave formation (arrows), confirming posterior myocardial infarction. STEMI, ST-segment elevation myocardial infarction

High-sensitivity troponin I peaked at 44,255 pg/mL (<16 pg/mL). Routine laboratory investigations were otherwise unremarkable, including a hemoglobin level of 13.2 g/dL (12.0-16.0 g/dL), leukocyte count of 7.8 × 10⁹/L (4.0-10.0 × 10⁹/L), platelet count of 265 × 10⁹/L (150-400 × 10⁹/L), serum creatinine of 0.72 mg/dL (0.50-1.10 mg/dL), C-reactive protein of 2.1 mg/L (<5 mg/L), and D-dimer level of 300 ng/mL (<500 ng/mL) (Table 1).

**Table 1:** Patient laboratory findings

Laboratory investigations	Patient findings	Normal values
Troponin I (pg/mL)	44,255	<16
Hemoglobin (g/dL)	13.2	12.0-16.0
Leukocyte count (10⁹/L)	7.8	4.0-10.0
Platelet count (10⁹/L)	265	150-400
Serum creatinine (mg/dL)	0.72	0.50-1.10
C-reactive protein (mg/L)	2.1	<5
D-dimer (ng/mL)	300	<500

Emergency coronary angiography was performed via right radial access using a 6-Fr diagnostic catheter, demonstrating total occlusion of the second obtuse marginal branch with diffuse luminal irregularity and contrast staining highly suggestive of Saw type I SCAD (Figure 2). The left coronary system was subsequently engaged with a 6-Fr extra-backup guiding catheter. A soft 0.014-inch workhorse coronary guidewire was carefully advanced toward the obtuse marginal lesion. Shortly after minimal contrast injection during guidewire manipulation, abrupt retrograde propagation of dissection occurred, with contrast staining extending from the LMCA into both the proximal LAD and LCx.

**Figure 2:**
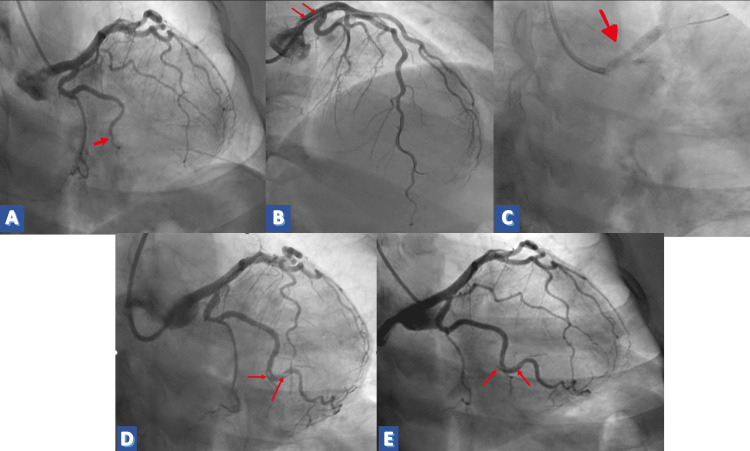
Coronary angiography and bailout management (A) Right anterior oblique caudal view demonstrating diffuse luminal irregularity of the marginal artery with TIMI 0 flow, suggestive of SCAD (arrow). (B) Left anterior oblique cranial view showing iatrogenic dissection extending from the LMCA into the proximal left anterior descending and circumflex arteries (arrow). (C) Contrast staining involving the left main bifurcation following dissection propagation (arrow). (D) Final angiographic result after bailout left main and circumflex stenting with restoration of TIMI 3 flow (arrows). (E) Follow-up angiography at three weeks demonstrating partial spontaneous healing of the marginal artery lesion with patent stents (arrows). LMCA, left main coronary artery; SCAD, spontaneous coronary artery dissection; TIMI, thrombolysis in myocardial infarction

During an attempt to cross the culprit lesion with a 0.014-inch guidewire, contrast injection triggered an acute iatrogenic dissection extending from the LMCA into the proximal LAD and LCx arteries. Subsequent contrast staining involving the left main bifurcation suggested severe flow impairment (Figure 2). The patient rapidly developed chest pain and hemodynamic collapse. Systolic blood pressure fell to 70 mmHg, requiring norepinephrine support.

An immediate bailout percutaneous coronary intervention (PCI) was undertaken. The left coronary system was engaged with a 6-Fr EBU 3.5 guiding catheter (Medtronic plc, Minneapolis, MN, USA). A Versaturn F guidewire (Abbott Laboratories, Abbott Park, IL, USA) was advanced into the distal LAD, and emergency bailout crossover stenting from the LMCA to the LAD was performed using a 4.0 × 24 mm SYNERGY MEGATRON drug-eluting stent (Boston Scientific Corporation, Marlborough, MA, USA), deployed at 12 atm without predilatation. Because of persistent compromise of the circumflex ostium, a second Versaturn F guidewire was advanced into the obtuse marginal branch, followed by predilatation of the proximal circumflex artery with a 3.0 × 15 mm EUPHORA SC balloon (Medtronic plc). Bailout T and small protrusion (TAP) stenting of the circumflex ostium was then performed using a 3.0 × 8 mm SYNERGY XD drug-eluting stent (Boston Scientific Corporation). Final optimization included kissing balloon inflation and proximal left main post-dilatation with a 4.5 × 12 mm NC EUPHORA balloon, achieving restoration of thrombolysis in myocardial infarction (TIMI) 3 flow with an optimal angiographic result (Figure 2). The marginal lesion demonstrated spontaneous reperfusion and was therefore managed conservatively. Intravascular imaging was not performed because of hemodynamic instability associated with left main compromise.

Echocardiography showed a left ventricular ejection fraction of 50% with inferolateral and anterolateral akinesia. The patient was discharged on dual antiplatelet therapy, beta-blocker therapy, and high-intensity statin therapy.

Repeat angiography performed three weeks later for recurrent chest pain without biomarker elevation demonstrated partial spontaneous healing of the marginal lesion with patent left main and circumflex stents (Figure 2). Coronary computed tomography angiography and fibromuscular dysplasia screening were scheduled during follow-up, but the patient preferred to defer additional investigations and continue clinical surveillance. At the six-month follow-up, the patient remained asymptomatic without recurrent ischemic events.

## Discussion

SCAD is a disruption of the coronary arterial wall layers due to an intimal tear. It predominantly affects younger women and is strongly associated with underlying arteriopathies, hormonal influences, and systemic inflammatory conditions. Many reviews emphasize that PCI in SCAD is associated with high rates of procedural complications and technical failure, particularly iatrogenic dissection propagation and extension. This reflects inherent arterial wall vulnerability and a tendency for extension with minimal instrumentation [4,5].

In the present case, guidewire manipulation and contrast injection were followed by an acute iatrogenic LMCA dissection extending into the proximal left anterior descending and circumflex arteries, resulting in hemodynamic collapse. Although the exact mechanism cannot be definitively ascertained, underlying arterial wall fragility in suspected SCAD likely facilitated dissection propagation during coronary instrumentation. Repeated contrast injections through a left main guiding catheter during guidewire manipulation of the distal lesion further contributed to hydraulic propagation of dissection into the left main bifurcation.

Several comparable cases of extensive iatrogenic left main dissection have been reported, although reports specifically occurring in the setting of SCAD remain exceedingly rare. Lao et al. described a patient who developed iatrogenic left main bifurcation dissection extending into both the LAD and circumflex arteries during PCI, resulting in impending vessel closure and hemodynamic compromise [6]. The lesion was successfully treated using immediate bailout TAP stenting of the left main bifurcation with a favorable long-term outcome. Similarly, Murthy et al. reported an extensive iatrogenic aorto-coronary dissection involving the left main, LAD, and LCx arteries during transradial PCI [7]. Intravascular ultrasound-guided bailout stenting of the left main and circumflex arteries restored TIMI 3 flow and stabilized the patient without the need for emergency surgery. Cepas-Guillén et al. reported a multivessel SCAD involving the LMCA, describing spontaneous dissections of the left main, LAD, and ramus intermedius arteries in a young woman presenting with STEMI [8]. Treatment consisted of left main-to-LAD crossover stenting because of ongoing ischemia and left main compromise. The circumflex artery did not require additional bifurcation rescue stenting; therefore, no TAP or culotte strategy was performed.

Compared with these reports, our case is distinguished by the abrupt retrograde propagation of dissection from a distal obtuse marginal SCAD after minimal contrast injection and coronary manipulation. Our case was particularly complex because it required emergency left main bifurcation rescue PCI with initial crossover stenting from the LMCA to the LAD, followed by bailout TAP stenting of the LCx ostium due to persistent side-branch compromise after main-vessel stenting. In addition, the need for emergent left main bifurcation rescue PCI using crossover LMCA-to-LAD stenting followed by TAP stenting of the LCx illustrates the technical complexity and management challenges associated with extensive SCAD-related iatrogenic propagation.

Once the left main flow became compromised, with ensuing hemodynamic collapse, conservative management was no longer feasible, and emergency bailout stenting became mandatory. Restoration of TIMI 3 flow rapidly stabilized the patient, while the original marginal lesion was intentionally left untreated. Conservative management is generally preferred whenever clinically feasible, given the high rate of spontaneous healing and the risk of procedural complications during PCI [9].

Follow-up angiography three weeks later demonstrated spontaneous healing of the marginal lesion, supporting current recommendations favoring conservative management of stable SCAD lesions whenever possible [10].

This case highlights the importance of early recognition of angiographic findings suggestive of SCAD and the need to minimize coronary instrumentation in this setting. When a catastrophic left main compromise occurs despite these precautions, rapid bailout stenting may be lifesaving.

The absence of intracoronary imaging represents the principal limitation of this report, precluding definitive confirmation of SCAD and its exact mechanism of propagation. However, optical coherence tomography and intravascular ultrasound were deferred because of profound hemodynamic instability during left main compromise.

## Conclusions

This case illustrates the potential for catastrophic iatrogenic left main dissection during coronary intervention in patients with angiographic findings suggestive of SCAD. Iatrogenic dissection in this setting may be amplified by hydraulic forces generated during contrast injection, particularly in vessels already weakened by SCAD. Early recognition of this entity should prompt a cautious interventional strategy with minimal coronary instrumentation whenever possible. When left main compromise occurs despite these precautions, rapid bailout stenting may be lifesaving, while the underlying SCAD lesion can often be managed conservatively with favorable spontaneous healing.

## References

[1] Brunton N, Best PJ, Skelding KA, Cendrowski EE (2024). Spontaneous coronary artery dissection (SCAD) from an interventionalist perspective. Curr Cardiol Rep.

[2] Adlam D, Alfonso F, Maas A, Vrints C (2018). European Society of Cardiology, acute cardiovascular care association, SCAD study group: a position paper on spontaneous coronary artery dissection. Eur Heart J.

[3] Sural S, Aslan V, Avcı G (2026). A dual catastrophe in the left main coronary artery: iatrogenic dissection and dislodgement of the fully deployed stent. BMC Cardiovasc Disord.

[4] Alfonso F, García-Guimaraes M, Bastante T, de la Cuerda F, Antuña P, Cuesta J, Rivero F (2018). Spontaneous coronary artery dissection: from expert consensus statements to evidence-based medicine. J Thorac Dis.

[5] Hayes SN, Kim ES, Saw J (2018). Spontaneous coronary artery dissection: current state of the science: a scientific statement from the American Heart Association. Circulation.

[6] Lao EP, Nie SP, Ma CS (2013). Immediate bail-out TAP-stenting for the treatment of iatrogenic aortocoronary dissection involving left main bifurcation. J Geriatr Cardiol.

[7] Murthy A, Singh A, Tuohy ER 4th (2014). Iatrogenic aorto-coronary dissection successfully treated with IVUS guided unprotected left main stenting: case report and review of literature. Cardiol Res.

[8] Cepas-Guillén PL, Flores-Umanzor EJ, Sabate M, Masotti M (2019). Multivessel spontaneous coronary artery dissection involving the left main coronary artery: a case report. Eur Heart J Case Rep.

[9] Lobo AS, Cantu SM, Sharkey SW (2019). Revascularization in patients with spontaneous coronary artery dissection and ST-segment elevation myocardial infarction. J Am Coll Cardiol.

[10] Geeslin D, Addington M, Trotman D (2025). Beyond the incision: understanding catheter induced coronary artery dissection. Curr Treat Options Cardio Med.

